# Burnout Trends Among US Health Care Workers

**DOI:** 10.1001/jamanetworkopen.2025.5954

**Published:** 2025-04-21

**Authors:** David C. Mohr, Shereef Elnahal, Maureen L. Marks, Ryan Derickson, Katerine Osatuke

**Affiliations:** 1 National Center for Organization Development, Veterans Health Administration, Mason, Ohio; 2 Department of Health Law, Policy & Management, Boston University School of Public Health, Boston, Massachusetts; 3 Undersecretary for Health Office, Veterans Health Administration, Washington, DC; 4 Department of Psychology, Miami University, Oxford, Ohio

## Abstract

**Importance:**

Burnout among health care workers is a widespread concern in health care both before and since the COVID-19 pandemic, yet little is known about health care workers’ burnout levels across occupations and settings.

**Objective:**

To examine trends in burnout and professional stress reported among health care workers working at the US Veterans Health Administration (VHA) and identify occupations that experienced notable changes and the factors associated with changes.

**Design, Setting, and Participants:**

This survey study used a retrospective cohort design grouped by key factors associated with burnout and professional stress. Responses to an annual organization-wide survey at 140 medical centers from 2018 to 2023 were used.

**Exposures:**

Respondents self-reported on 2 burnout items (ie, “I feel burned out from my work” and “I worry that this job is hardening me emotionally”) from the Maslach Burnout Inventory during all study years and professional stress (moderate or lower vs high or extreme) from COVID-19 from 2020 to 2023.

**Main Outcome and Measures:**

Trends by occupation, telework status, and geographic region were examined, as well as the general pattern over time and the change in burnout and stress rates in the years following the start of the pandemic.

**Results:**

In 2018, the sample was 71.6% female, with an estimated mean (SD) age of 46.31 (12.11) years, and estimated mean (SD) VA tenure of 8.54 (7.33) years. Totals of health care worker respondents identified from 140 medical centers ranged from 123 271 in 2018 to 169 448 in 2023. Annual burnout rates were 30.4% for 2018, 31.3% for 2019, 30.9% for 2020, 35.4% for 2021, 39.8% for 2022, and 35.4% for 2023. Rates of professional stress from COVID-19 were 32.0% for 2020, 26.9% for 2021, 29.2% for 2022, and 21.4% for 2023. Both measures showed a decrease following the official public health emergency ending in 2023. Primary care physicians reported the highest burnout levels compared with other service areas, ranging from 46.2% in 2018 to 57.6% in 2022. Several service areas saw a relative increase of 10% or more in burnout between 2018 and 2023, with mental health, dental, and rehabilitation service employees reporting the highest increases in burnout rates over this time. Burnout levels for respondents who teleworked most of the time were lower than those for respondents who did not telework.

**Conclusions and Relevance:**

In this survey study of VHA health care workers, burnout and professional stress decreased on average following the pandemic, but burnout levels remain elevated compared with prepandemic levels. The VHA has made several efforts to reduce burnout and stress, and results showed some promise, but exploration of ways to reduce burnout to prepandemic levels is needed.

## Key Points

**Question:**

How have levels of burnout changed before, during, and after the COVID-19 pandemic among health care workers at the Veterans Health Administration (VHA)?

**Findings:**

In this 6-year survey study of VHA health care workers (ranging from 123 271 in 2018 to 169 448 in 2023), burnout levels generally decreased following the pandemic but remain high compared with prepandemic levels.

**Meaning:**

These findings suggest the need to explore ways to decrease burnout levels to prepandemic levels.

## Teaser

This survey study examines trends in burnout and stress before, during, and after the COVID-19 pandemic among the health care workforce at the Veterans Health Administration.

## Introduction

Burnout among health care workers and burnout solutions have been topics of great interest and the subject of a call to action in US health care prior to the COVID-19 pandemic.^1,2^ Driven in part by the COVID-19 pandemic concerns and by changes in workforce practices,^3,4,5^ including greater telework flexibility^6^ and telemedicine,^7^ the pandemic exacerbated existing challenges and introduced new ones, leading to health care workers considering earlier retirement, changing job roles, or “quiet quitting” (ie, covert disengagement).^8,9^ This increased the imperative for organizations to do more to support employees, make work more manageable, and continue to deliver patient care. The confluence of factors helped give rise to solutions to promote employee well-being^10,11^ at both individual-level interventions, such as increasing resilience,^12,13^ and system-level interventions, such as shifting the work demands, staffing, or leadership practices.^14,15,16^

While some research suggests that burnout levels in health care increased during the pandemic and then started to return to prepandemic levels,^17,18^ other work shows burnout levels remaining unchanged or higher.^19^ Questions regarding how health care workers are doing following the disruptions in clinical care and daily life remain. For organizations, questions exist on whether the activities and programs conceived and offered during this time will become the usual practice, intensify, or cease. Further, employees wonder if changes made to increase work-life flexibility and offer more ways of delivering care will continue and what practice changes intended to make work easier will remain.

This analysis focuses on the Veterans Health Administration (VHA), the largest integrated health system in the United States. The VHA employs more than 419 000 individuals, including approximately 278 000 health care workers across 172 medical centers. VHA locations can be found broadly across the US based on population density and the needs of local veterans and operate within a broad range of metropolitan to very rural areas. Research within the organization has often focused on burnout among physicians,^1,20^ with less focus on other health care occupations and contextual settings that may influence burnout.

The VHA has implemented several system-level programs to revise organizational practices and policies to increase individual resilience and to reduce or mitigate burnout. Prior to the pandemic, the VHA engaged in efforts to improve processes for assessing and responding to burnout, including incorporating greater use of data to understand employee experience using the administration of an all-employee survey (AES). During the course of the COVID-19 pandemic and afterward, the VHA implemented many programs to continue to provide high-quality care for patients, mitigate employee burnout, and improve employee well-being, such as introducing whole health practices to employees, reducing workload by hiring more employees, and increasing options for telehealth and telework. The impact of these incipient changes on burnout has not been studied. Our study also sought to examine health care workers’ burnout levels across occupations and settings.

The present analysis uses data from the AES to examine trends in burnout and stress, particularly focused on 1 to 2 years before, during, and after the COVID-19 public health declaration. The high survey participation rate from the occupationally and demographically diverse VHA health care workforce across the US provides insights into how a large portion of US health care workers’ experiences changed during a recent multiyear period. We present findings in a descriptive manner and test for differences by individual- and system-level factors.

## Methods

### Data Sources

The study design was a longitudinal cross-sectional survey analysis. Data were obtained from the annual AES from June 4 to 25, 2018; June 3 to 24, 2019; September 14 to October 5, 2020; June 7 to 28, 2021; June 6 to 28, 2022; and June 5 to 27, 2023. AES data are routinely used for supporting strategic planning and providing feedback.^21,22^ The online anonymous survey followed best practices by the American Association for Public Opinion Research (AAPOR). The core survey consists of approximately 60 items about workplace experiences and perceptions and asks for information on the demographic characteristics of respondents. This analysis focused on burnout and stress from COVID-19 at the individual level in the VHA workforce. The reporting guideline for Strengthening the Reporting of Observational Studies in Epidemiology (STROBE) was also considered. The University of Cincinnati and Cincinnati Veterans Affairs Medical Center Institutional Review Board determined that this secondary analysis was not human research and did not require approval or informed consent.

### Sample

We restricted our sample to include health care workers based on self-identification. The number of health care worker respondents per year from 140 medical centers ranged from 123 271 in 2018 to 169 448 in 2023 and reflects an increase in total staff and in response rates (eAppendix 1 in Supplement 1). Internal quality control using human resources records has shown that self-reported demographic characteristics (eg, age, gender, race and ethnicity) are relatively proportionate to the organization’s population.

### Measures

We computed burnout for each respondent using 2 Maslach Burnout Inventory^23^ items representing emotional exhaustion (“I feel burned out from my work”) and depersonalization (“I worry that this job is hardening me emotionally”). Respondents rated these items from “never” to “every day” using a 7-point frequency scale. Respondents who reported experiencing either symptom once a week or more were coded as experiencing burnout (1); a value of 0 was coded for fewer occurrences.^24,25^ COVID-19 professional stress was assessed using the originally developed item, “How much stress has the COVID-19 pandemic added to your day-to-day work?” Response options included none, minimal, moderate, high, and extreme. We created a dichotomous variable to indicate high stress based on selection of the 2 highest categories (1) to compare with those reporting moderate or lower stress levels (0). The item was asked in 2020 through 2023.

Two broad sets of measures were examined for trending against burnout. Individual-level variables included occupation, which was a choice from 46 health care roles. Telework, which can influence burnout,^26^ was examined based on the item, “How often do you telework?” We created variables as none (do not telework or telework on occasion), partial (<1 or 1-2 days/week), and majority (3-4 or 5 days/week). Respondents also indicated the primary service they provided, which may influence burnout,^27^ including as many as 26 choices (eAppendix 2 in Supplement 1). All 3 measures were from AES responses. System-level variables were used to represent the context of the respondent. Geographic division consisted of 9 areas defined by the US Census Bureau and assigned based on state locations of the medical center.

### Statistical Analysis

Analyses first consisted of scoring burnout and professional stress for each employee and coding for telework. Because the purpose was a descriptive report of prevalence trends at an aggregate level, we did not adjust for respondent demographic characteristics that may influence burnout, although we recognize that burnout is a complex phenomenon influenced by many factors. Burnout and professional stress were computed at an individual level and examined for variation across measures by year. We did not substitute missing values. We computed inferential statistics to assess the trend in outcomes based on individual (occupation and telework) and region using 1-way analysis of variance with a Tukey adjustment. We tested the difference between (1) burnout before (2018) and after (2023) the pandemic and (2) the year the pandemic ended (2022) and following the pandemic (2023). Analyses were conducted using SAS software, version 9.4 (SAS Institute Inc). Two-sided *P* < .05 indicated statistical significance.

## Results

The annual response rate ranged from 210 057 of 341 144 eligible respondents (61.6%) in 2018 to 293 164 of 394 646 (74.3%) in 2023; among these, the numbers of health care worker respondents ranged from 123 271 in 2018 to 169 448 in 2023. At medical centers, burnout ranged from 30.4% in 2018 to 39.8% in 2022, and high levels of professional stress ranged from 21.4% in 2023 to 29.2% in 2022. Burnout was relatively stable leading into the pandemic and during the first year (30.4% in 2018; 31.3% in 2019; 30.9% in 2020) with no significant difference but then increased significantly in 2021 (35.4%) and 2022 (39.8%), followed by a decrease during the time after the official COVID-19 public health emergency ended (35.4%) in May 2023 (*P* < .001). Looking across years, relative year-to-year change was 3.0% for 2018 to 2019, −1.9% for 2019 to 2020, 15.6% for 2020 to 2021, 13.0% for 2021 to 2022, and −11.5% for 2022 to 2023. Burnout was 16.4% relatively higher in the last year compared with the first year of the study. The trend for high professional stress was significant in all comparisons between years with the highest rate in 2020 when the pandemic was declared (32.0%), followed by a decline (26.9%) in 2021, a small increase (29.2%) in 2022, and then a notable decrease (21.4%) after the pandemic (*P* < .001). Looking across years, the relative year-to-year change was −15.8% (2020 to 2021), 8.3% (2021 to 2022), and −26.6% (2022 to 2023).

### Occupations

Among occupations, primary care physicians consistently had the highest burnout throughout the time frame evaluated, with values ranging from 46.2% in 2018 to 57.6% in 2022 (Table 1). Several occupations saw burnout levels increase by 10% or more between 2018 and 2023, including dentists (from 26.7% to 41.7%), psychologists (from 34.1% to 47.6%), dietitians (from 26.3% to 38.6%), and optometrists (from 36.9% to 46.7%). No decreases in burnout of 5% or greater were seen between 2018 to 2023. In contrast, 20 occupations showed a decrease in COVID-19 stress by 10% or more (Table 2). Example occupations with the largest decreases were among registered nurses (eg, level V, from 45.6% in 2020 to 24.2% in 2023), licensed practical nurses (from 36.0% in 2020 to 24.7% in 2023), audiologists (from 35.0% in 2020 to 15.5% in 2023), optometrists (from 29.9% in 2020 to 11.0% in 2023), psychologists (from 31.9% in 2020 to 14.3% in 2023), and physical therapists (from 27.9% in 2020 to 14.3% in 2023).

**Table 1.** Burnout Trends by Occupation in the Veterans Health Administration

Occupation	Survey year, % of respondents					
	2018	2019	2020	2021	2022	2023
Physician						
Primary care	46.2	47.0	45.6	53.8	57.6	56.5
Surgery	25.8	24.2	20.7	26.5	28.5	27.9
Psychiatry	33.3	35.9	35.7	43.9	46.7	45.0
Anesthesiology	25.4	23.6	20.9	26.8	27.1	23.1
Medicine	24.7	24.5	23.4	29.7	32.6	29.7
All other	24.3	26.6	23.3	29.4	34.9	32.3
Dentist	26.7	33.6	28.7	38.0	42.9	41.7
Certified registered nurse anesthetist	27.5	27.3	24.7	28.8	31.3	27.6
Optometrist	36.9	35.7	32.0	39.3	47.0	46.7
Physician assistant	30.8	33.8	32.7	36.9	41.3	39.7
Nurse practitioner	31.5	32.6	32.0	37.7	43.1	39.6
Registered nurse						
Level I	31.3	30.5	30.6	35.0	40.3	34.0
Level II	29.6	30.9	30.4	34.3	40.0	34.0
Level III	28.1	28.8	30.4	34.1	38.1	32.6
Level IV	30.0	32.0	34.6	40.7	43.3	37.5
Level V	25.6	26.5	23.0	37.0	35.8	35.0
Licensed practical nurse	28.0	28.7	30.0	31.9	37.3	31.4
Nursing assistant	29.3	30.2	29.2	33.0	37.3	32.7
Pharmacist	37.2	38.8	35.8	43.2	49.8	45.0
Pharmacy technician	35.1	35.5	32.1	36.5	41.4	34.2
Psychologist	34.1	38.3	40.2	48.1	51.8	47.6
Social worker	29.7	30.7	30.9	36.0	40.3	36.3
Respiratory therapist	26.7	24.8	25.1	26.2	30.9	29.7
Other certified or licensed health care worker	30.8	32.3	28.6	34.0	39.4	34.0
Other noncertified or nonlicensed health care worker	29.4	29.4	28.0	31.1	36.5	34.1
Clinical laboratory employee	35.2	34.8	34.2	40.3	45.5	38.6
Diagnostic imaging technician	29.9	29.7	29.3	32.6	35.8	30.0
Health care technician	31.8	31.4	30.4	32.4	36.1	32.7
Dietitian and intern	26.3	28.0	28.4	37.3	42.0	38.6
Physical therapist	NA	NA	27.3	33.8	38.0	35.5
Recreational therapist	NA	NA	26.5	28.8	31.6	32.2
Occupational therapist	NA	NA	27.8	31.4	36.2	34.6
Audiologist	NA	NA	32.7	39.4	47.1	46.3

Abbreviation: NA, not available.

**Table 2.** COVID-19 Professional Stress Trends by Occupation in the Veterans Health Administration

Occupation	Survey year, % of respondents			
	2020	2021	2022	2023
Physician				
Primary care	29.4	24.1	27.5	22.4
Surgery	23.2	16.5	18.0	16.4
Psychiatry	26.2	19.3	20.7	16.7
Anesthesiology	34.6	28.6	23.9	22.1
Medicine	31.1	30.3	31.1	25.3
All other	26.8	24.1	26.1	20.6
Dentist	33.2	30.7	27.5	25.2
Certified registered nurse anesthetist	39.3	32.3	30.6	26.2
Optometrist	29.9	21.0	13.0	11.0
Physician assistant	23.0	17.9	21.4	16.7
Nurse practitioner	25.4	20.9	25.2	17.8
Registered nurse				
Level I	37.1	33.1	32.5	22.9
Level II	37.9	32.8	34.7	25.6
Level III	34.4	29.4	31.4	21.0
Level IV	43.5	38.4	35.2	20.3
Level V	45.6	35.2	40.2	24.2
Licensed practical nurse	36.0	29.1	33.5	24.7
Nursing assistant	39.6	34.7	40.6	32.6
Pharmacist	23.6	21.9	24.6	17.6
Pharmacy technician	30.2	24.1	26.5	19.9
Psychologist	31.9	23.1	22.2	14.3
Social worker	29.8	22.2	26.1	16.0
Respiratory therapist	37.4	38.4	38.6	30.8
Other certified or licensed health care worker	32.2	27.7	31.1	22.9
Other noncertified or nonlicensed health care worker	31.1	23.6	27.9	23.0
Clinical laboratory employee	33.2	30.9	34.6	26.6
Diagnostic imaging technician	34.3	27.0	28.8	21.9
Health care technician	33.8	26.1	29.9	22.0
Dietitian and intern	20.8	15.3	18.3	11.1
Physical therapist	27.9	21.3	20.7	14.3
Recreational therapist	34.5	33.0	39.5	27.3
Occupational therapist	27.1	21.7	21.7	15.9
Audiologist	35.0	22.4	20.4	15.5

### Telework

Regarding telework, the percentage of health care workers with limited telework increased from 8.4% in 2018 to 20.9% in 2023 and increased for those with majority telework from 3.4% in 2018 to 11.8% in 2023 (eFigure 1 in Supplement 1). Burnout levels for telework most of the time ranged between 26.2% in 2018 to 37.7% in 2022 compared with nontelework employees, whose burnout ranged between 30.5% in 2018 to 40.0% in 2022 (*P* < .001). For professional stress, employees teleworking on most days of the week had lower stress rates by year, while those using some telework had slightly higher stress levels and those with no telework had the highest stress levels (eFigure 2 in Supplement 1).

### Service Area

All service areas showed an increase in burnout between 2018 and 2023 (eTable 1 in Supplement 1). The largest increases in burnout were seen in dental (from 30.7% to 39.6%), mental health (from 30.4% to 38.2%), and rehabilitation service (from 27.1% to 34.1%) areas. When comparing the change between 2022 and 2023, all service areas showed a decrease, with larger decreases for emergency medicine (from 43.3% to 35.2%), laboratory and pathology medicine (from 46.6% to 39.0%), intensive care unit (ICU) (from 39.7% to 32.5%), and acute care (from 44.5% to 37.3%).

Employees working in the ICU (48.6%), emergency medicine (45.8%), acute care (40.0%), and community living centers (39.6%) reported the highest stress levels at the start of the pandemic. All service areas, however, saw a decrease in professional work stress from 2020 to 2023, with optometry (16.3%), administrative areas (14.1%), emergency medicine (13.2%), and ICU (12.9%) showing the largest decreases (eTable 2 in Supplement 1).

### Geographic Regions

Trends in burnout and professional stress by geographic region are shown in the Figure. Geographic regions with the largest increases in burnout between 2018 and 2023 included the Pacific (7.0%), West North Central (6.5%), New England (6.0%), and South Atlantic (5.7%) states. Employees in sites in the East South Central (6.6%), West North Central (6.1%), and Mountain (5.6%) states reported the largest decreases in burnout between 2022 and 2023 (eFigure 3 in Supplement 1). Employees in areas that experienced the largest decrease in professional stress between 2020 and 2023 included the West South Central (12.7% decrease), East North Central (12.2% decrease), and East South Central (11.7% decrease) states (eFigure 4 in Supplement 1).

**Figure.**
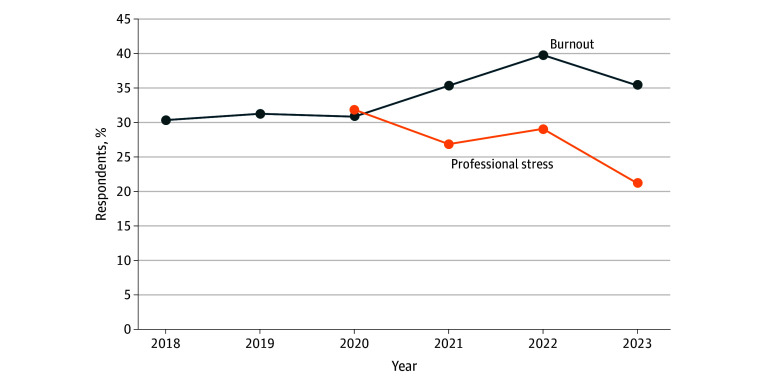
Trends in Burnout and COVID-19 Professional Stress Among Veterans Health Administration Health Care Workers by US Census Bureau Region One bar is equal to the mean burnout or stress for a given year.

## Discussion

This survey study examined how burnout and professional stress changed before, during, and after the COVID-19 pandemic among health care workers. The VHA saw burnout and professional stress decrease, on average, among tens of thousands of health care workers after the pandemic. We noticed burnout increased over time during the pandemic, while professional stress began to decline. This may be due to the initial set of shocks and uncertainty followed by greater stabilization that led to pandemic-related stress to decrease. Burnout showed a relative decrease of 1.9% in the first year of the pandemic, followed by much larger increases from 2020 to 2021 and 2021 to 2022. The reasons for this increase are unclear but may be due to challenges in community care coordination, facility budgets, staffing limitations, or a greater set of expectations among patients or pandemic-related fatigue.^28^ Despite the reduction that followed in 2023, burnout was 16.4% higher in 2023 compared with 2018. Compared with professional stress, this finding suggests that burnout may be slower to develop and slower to improve. Thus, focus on reducing burnout remains critical.

The effort-recovery model of work stress suggests that the mental, physical, emotional, and other resources employees expend to meet job demands lead to reductions in these resources; time away from work can assist with recovering those resources.^29^ Employees can experience burnout and recovery differently; some may show increases in emotional thriving right away, while others may need more time for recovery.^30^ Further time may be needed to allow employees recovery following the increased and chaotic job demands and challenges faced during the pandemic. Likewise, interventions intended to improve the workplace environment may take additional time to spread widely and impact complex workplace issues involving stress and burnout. Careful attention to burnout and recovery following the pandemic will be helpful to understand whether an observed decrease in burnout will become a trend.

A robust method for regularly surveying employees about their experiences of organizational health issues and continuous efforts to improve working conditions may help address burnout. In the last few years, the VHA has encouraged and supported various initiatives, such as refining workplace roles for greater efficiency, having flexibility in work and meeting hours, expanding employee-facing well-being resources along with a chief well-being officer role, and increasing health care staff to both support employees and patient care delivery.

VHA health care workers generally reported a decrease in burnout following the pandemic. It is unclear to what extent this reflects a broader change in the health care landscape and/or the influence of a proactive stance to address burnout. In comparison, surveys on physician burnout show notable changes in physician burnout during a similar study time frame with rates of 43.9% in 2017 and 38.2% in 2020, increasing to 62.8% in 2021, and then declining to 53.0% in 2022 and 48.2% in 2023.^31,32,33^ In contrast, a study looking at the broader health care workforce reported an increase in burnout of 67.9% in 2018 to 79.1% in 2022.^34^ Other work^35^ has shown nurses reporting increased emotional exhaustion, while physicians experienced initial decreases and then a sharp increase during the pandemic, making the need to understand changes and prevention strategies important. Despite whatever reductions have occurred in recent years, burnout levels are higher today than 5 years ago.

While previous research in the VHA and elsewhere mainly focused on physicians, our data suggest that this single scope of attention is insufficient. Looking across occupations, the high burnout rates among mental health professionals, including psychiatrists, psychologists, and social workers, underlies the need to direct more focus to these occupations and attend to occupation-specific causes of burnout and stress. Further, burnout rates among nurses were much higher than several of the physician specialties, highlighting the need for tailoring approaches to specific occupations. For example, offering alternative work schedules may be especially beneficial for nurses. There are notable differences along with underlying causes among health care professions on burnout that may influence the improvement change selected and its effectiveness.^36^ While the pandemic may have been declared over, many individuals are experiencing long-term effects^37^ that may impact their experiences as patients and health care workers.

It may be just as important to consider the service area when developing interventions. Health care workers in the emergency department encounter different stressors and work demands than those in the primary care or surgical setting. Thus, while a one-size-fits-all approach may address many drivers of burnout, specific attention should be given to addressing both the nature and the setting of health care work. It is also important to avoid adding to levels of burnout or stress when introducing changes intended to improve work life.

### Limitations

Our study has several limitations. While we examined how stress and burnout changed over time against specific health care worker occupations, geographic areas, and clinical specialty areas, we were unable to connect specific practice changes to a reduction in stress and burnout. While a broad approach to improving the workplace may lead to multiple benefits, it is not always clear which activities had the most impact, which makes it harder to understand which practices, ranging from low-resource to very-high-resource intensive, may be most cost-effective and useful for realizing improvements. While we had a large sample of respondents across occupational roles, findings may not generalize to other health care settings or different occupational roles, although trends may allow comparison and insights at a general level for other institutions. While the pandemic period demonstrated higher levels of burnout, as we expected, unique experiences of employees and challenges individually experienced both at work and in personal lives may confound this observed effect, especially if other systematic challenges exist that are unique to the VHA. Because we have some degree of overlap among unique respondents across years, we have been cautious in interpreting results, as significant differences may be influenced by some cohort of similar employee responses over time rather than reflect a true change in the population. Qualitative research may offer a way of better understanding the lived experiences of our health care workers. Our study was limited in its ability to trend individual data and instead relied on cohorts based on broadly defined criteria, such as occupation and region. We did not adjust for sociodemographic characteristics in computing burnout prevalence, which may have important influences.^38,39^ The choice of which items to use to measure burnout can also lead to different estimates of prevalence rates,^40,41,42^ which can make benchmarking and comparisons challenging to understand over time.^43^

## Conclusions

In this survey study of the VHA clinical workforce, we saw a rapid increase in burnout since the start of the pandemic, followed by a notable decrease in burnout the year after the pandemic but at a level still elevated compared with the prepandemic environment. As health care organizations continue in the postpandemic phase, it remains important to find ways to continue and sustain improvements in health care worker well-being and reduce burnout at work. The VHA experience and the data we report herein suggest that such improvements are possible. Toward these ends, we believe there is great value for health care workers and health care systems in documenting changes in burnout rates and studying the influence of proactive organizational efforts.

## Notes

Supplement 1. **eAppendix 1.** Number of Respondents Overall and by Clinical Occupation and Year **eAppendix 2.** Individual-Level Measures From the Survey **eTable 1.** Burnout by Primary Service Area Trends in the VHA **eTable 2.** COVID-19 Professional Stress Trends by Main Service Provided in the VHA **eFigure 1.** Burnout by Telework Trends in the VHA **eFigure 2.** COVID-19 Professional Stress Trends by Telework in the VHA **eFigure 3.** Burnout Trends by Geography in the VHA **eFigure 4.** Professional Stress Trends by Geography in the VHA

Supplement 2.
Data Sharing Statement
