# Supplementary material for: Burnout Trends Among US Health Care Workers
Source: JAMA Netw Open. 2025 Apr 21;8(4):e255954. doi: 10.1001/jamanetworkopen.2025.5954 (PMC12013355; doi:10.1001/jamanetworkopen.2025.5954)
Supplement: Supplement 1. — eAppendix 1. Number of Respondents Overall and by Clinical Occupation and Year eAppendix 2. Individual-Level Measures From the Survey eTable 1. Burnout by Primary Service Area Trends in the VHA eTable 2. COVID-19 Professional Stress Trends by Main Service Provided in the VHA eFigure 1. Burnout by Telework Trends in the VHA eFigure 2. COVID-19 Professional Stress Trends by Telework in the VHA eFigure 3. Burnout Trends by Geography in the VHA eFigure 4. Professional Stress Trends by Geography in the VHA [file jamanetwopen-e255954-s001.pdf]

Supplementary Online Content

Mohr DC, Elnahal S, Marks ML, Derickson R, Osatuke K. Burnout trends among US health care workers. *JAMA Netw Open.* 2025;8(4):e255954. doi:10.1001/jamanetworkopen.2025.5954

eAppendix 1. Number of Respondents Overall and by Clinical Occupation and Year

eAppendix 2. Individual-Level Measures From the Survey

eTable 1. Burnout by Primary Service Area Trends in the VHA

eTable 2. COVID-19 Professional Stress Trends by Main Service Provided in the VHA

eFigure 1. Burnout by Telework Trends in the VHA

eFigure 2. COVID-19 Professional Stress Trends by Telework in the VHA

eFigure 3. Burnout Trends by Geography in the VHA

eFigure 4. Professional Stress Trends by Geography in the VHA

This supplementary material has been provided by the authors to give readers additional information about their work.

© 2025 Mohr DC et al. *JAMA Network Open*.

eAppendix 1. Number of Respondents Overall and by Clinical Occupation and Year

Year	Total respondents	Possible respondents	Response rate	Health care respondents
2018	210057	341144	61.57%	123271
2019	224891	351723	63.94%	132614
2020	253394	364113	69.59%	151376
2021	251925	371341	67.84%	148772
2022	262120	375174	69.87%	152110
2023	293164	394646	74.29%	169448

***** Exact number of possible healthcare respondents unknown

© 2025 Mohr DC et al. *JAMA Network Open*.

eAppendix 2. Individual-Level Measures From the Survey

Stress.

How much stress has the COVID-19 pandemic added to your day-to-day work?

Response options: 1 = None, 2 = Minimal, 3 = Moderate, 4 = High, 5 = Extreme

Burnout

I feel burned out from my work

I worry that this job is hardening me emotionally

Response options: 0 = Never, 1 = A few times a year or less, 2 = Once a month or less, 3 = A few times a month, 4 = Once a week, 5 = A few times a week, 6 = Every day

Telework.

How often do you telework?

2018-2019	2020-2023
I do not telework[a]	I do not telework because I choose not to[a]
I telework on occasion[b]	I do not telework because I cannot perform my job duties from home[a]
I telework a majority of the time[c]	I do not telework because I have not been approved to do so even though I could perform my job duties from home[a]
I telework full-time[c]	I telework less than 1 day per week[b]
	I telework 1 - 2 days per week[b]
	I telework 3 - 4 days per week[c]
	I telework 5 days per week[c]

Note: [a] coded as “none”; [b] coded as “partial”; [c] coded as “majority”

States included in geographic regions

1. New England: Connecticut; Maine; Massachusetts; New Hampshire; Rhode Island; Vermont
2. Middle Atlantic: New Jersey; New York; Pennsylvania
3. East North Central: Indiana; Illinois; Michigan; Ohio; Wisconsin
4. West North Central: Iowa; Kansas; Minnesota; Missouri; Nebraska; North Dakota; South Dakota
5. South Atlantic: Delaware; District of Columbia; Florida; Georgia; Maryland; North Carolina; South Carolina; Virginia; West Virginia
6. East South Central: Alabama; Kentucky; Mississippi; Tennessee
7. West South Central: Arkansas; Louisiana; Oklahoma; Texas
8. Mountain: Arizona; Colorado; Idaho; Montana; New Mexico; Nevada; Utah; Wyoming
9. Pacific: Alaska; California; Hawaii; Oregon; Washington

© 2025 Mohr DC et al. *JAMA Network Open*.

Appendix C.

eTable 1. Burnout by Primary Service Area Trends in the VHA

Primary Service	2018	2019	2020	2021	2022	2023
Administrative	28.4%	29.2%	30.4%	35.0%	38.9%	34.5%
Dental	30.7%	33.5%	30.2%	38.3%	43.9%	39.6%
Emergency medicine	30.6%	34.5%	29.7%	34.5%	43.3%	35.2%
Home/community care	26.5%	28.0%	26.5%	29.8%	34.1%	32.5%
Imaging	27.6%	27.8%	26.7%	30.0%	33.8%	29.6%
Intensive/critical care unit	27.3%	28.6%	30.4%	31.3%	39.7%	32.5%
Laboratory and pathology	34.6%	34.2%	35.6%	40.5%	46.6%	39.0%
Medical specialty	27.5%	27.7%	25.6%	31.0%	34.3%	30.9%
Mental health	30.5%	32.3%	32.8%	39.1%	42.5%	38.2%
Community Living Center	31.6%	32.7%	30.7%	36.7%	38.9%	36.3%
Pharmacy	35.3%	37.0%	33.8%	39.6%	46.3%	40.0%
Primary care	36.5%	37.1%	38.8%	43.2%	47.2%	42.5%
Rehabilitation services	27.1%	27.4%	25.5%	30.1%	35.5%	34.1%
Spinal cord injury	29.9%	31.9%	29.8%	34.9%	43.3%	36.5%
Surgery, anesthesiology, or surgical specialty	27.1%	26.1%	24.8%	29.3%	33.1%	28.3%
Acute care inpatient	31.2%	32.4%	31.2%	36.4%	44.5%	37.3%
Optometry			32.0%	34.6%	42.4%	41.7%
Other clinical service	27.8%	28.6%	27.7%	30.6%	35.0%	30.8%

© 2025 Mohr DC et al. *JAMA Network Open*.

eTable 2. COVID-19 Professional Stress Trends by Main Service Provided in the VHA

Primary service	2020	2021	2022	2023
Administrative	32.2%	25.8%	26.9%	18.1%
Dental	36.7%	31.7%	31.5%	26.2%
Emergency medicine	45.8%	42.9%	45.5%	32.6%
Home/community care	24.9%	20.0%	24.6%	17.6%
Imaging	30.9%	25.0%	26.0%	20.2%
Intensive/critical care unit	48.6%	48.5%	47.1%	35.7%
Laboratory and pathology	35.1%	32.0%	36.7%	27.3%
Medical specialty	29.5%	25.0%	27.3%	20.9%
Mental health	30.1%	23.7%	26.5%	18.5%
Community Living Center	39.6%	36.5%	42.1%	33.9%
Pharmacy	26.2%	23.1%	26.2%	19.1%
Primary care	33.3%	26.9%	30.1%	21.3%
Rehabilitation services	29.7%	22.1%	24.8%	17.4%
Spinal cord injury	34.8%	30.6%	33.2%	26.8%
Surgery, anesthesiology, or surgical specialty	33.7%	25.2%	26.2%	20.5%
Acute care inpatient	40.0%	38.4%	39.7%	29.6%
Optometry	29.9%	22.3%	17.5%	13.6%
Other clinical service	30.0%	23.4%	26.6%	19.2%

© 2025 Mohr DC et al. *JAMA Network Open*.

eFigure 1. Burnout by Telework Trends in the VHA

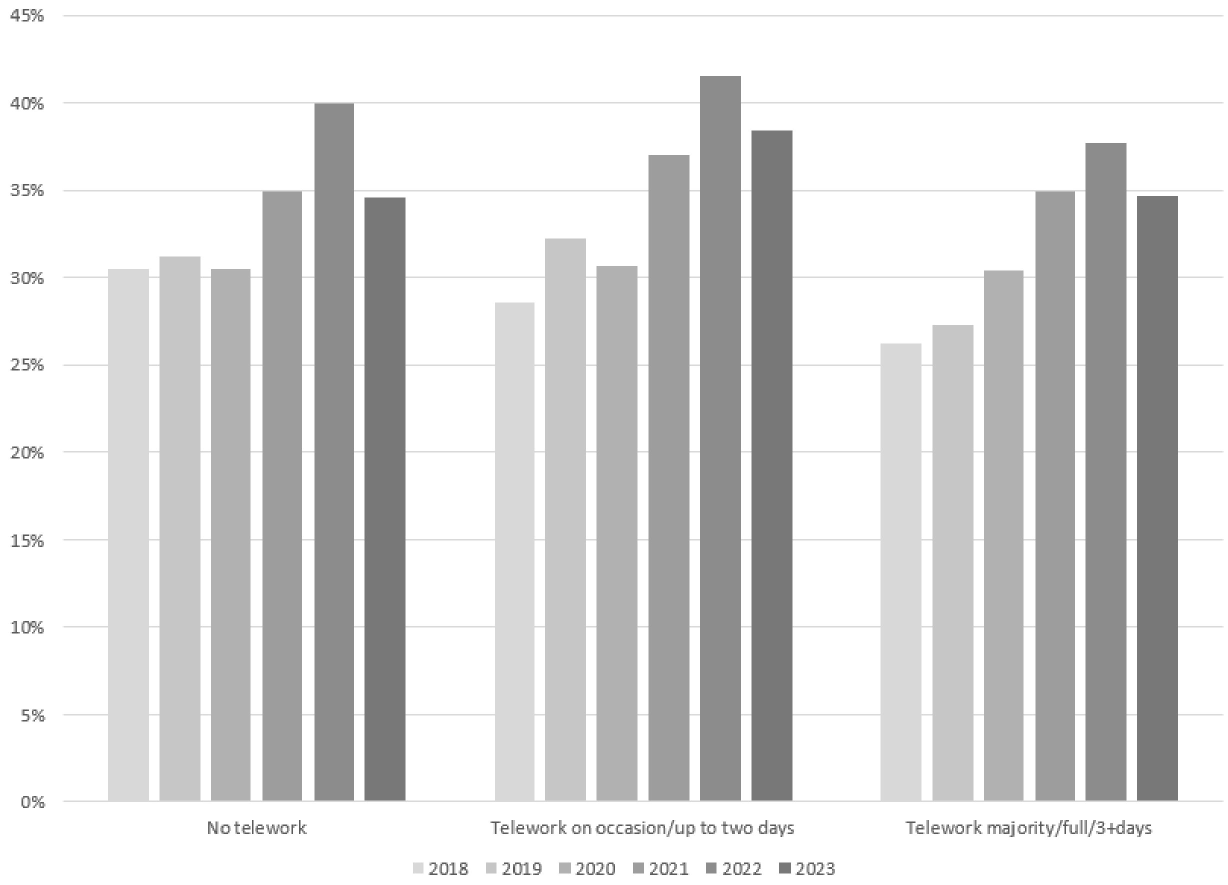

© 2025 Mohr DC et al. *JAMA Network Open*.

eFigure 2. COVID-19 Professional Stress Trends by Telework in the VHA

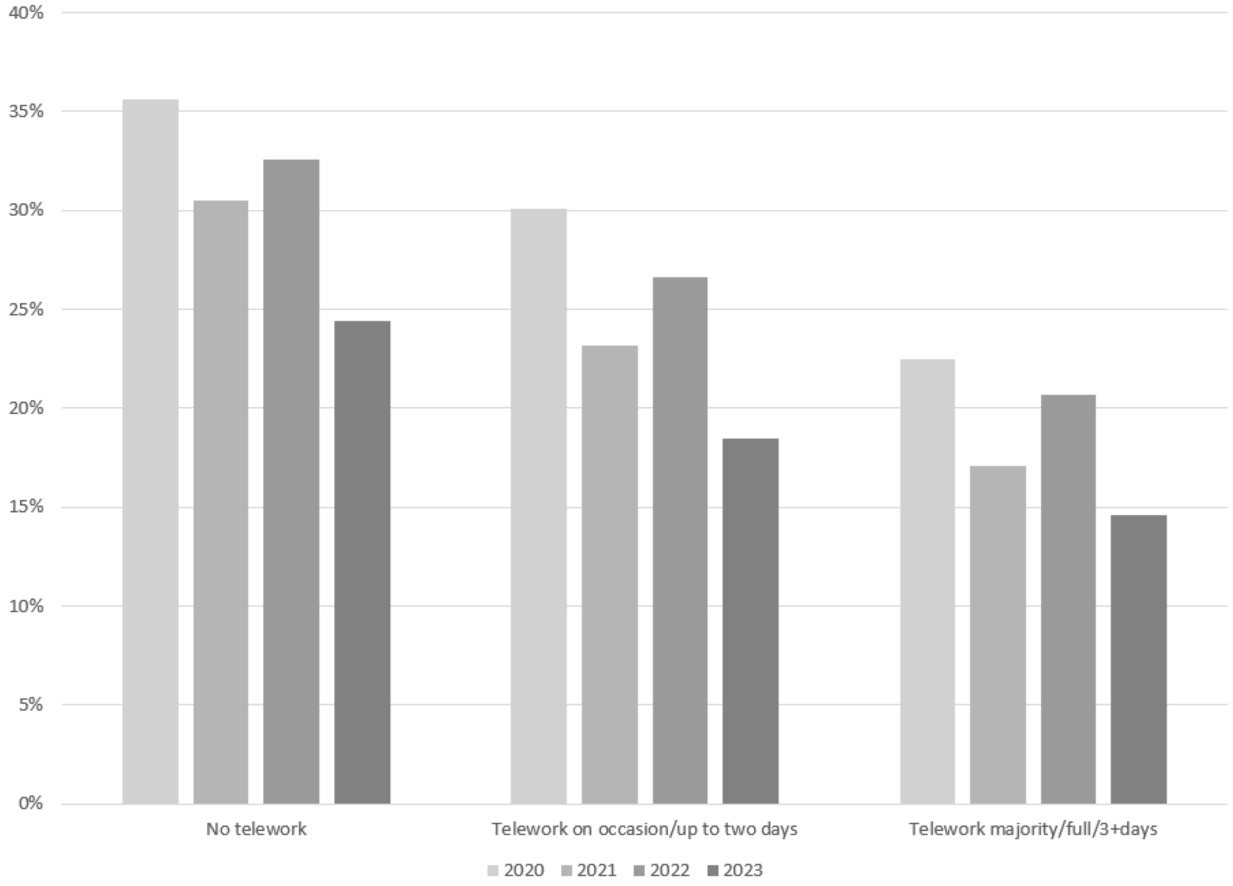

© 2025 Mohr DC et al. *JAMA Network Open*.

eFigure 3. Burnout Trends by Geography in the VHA

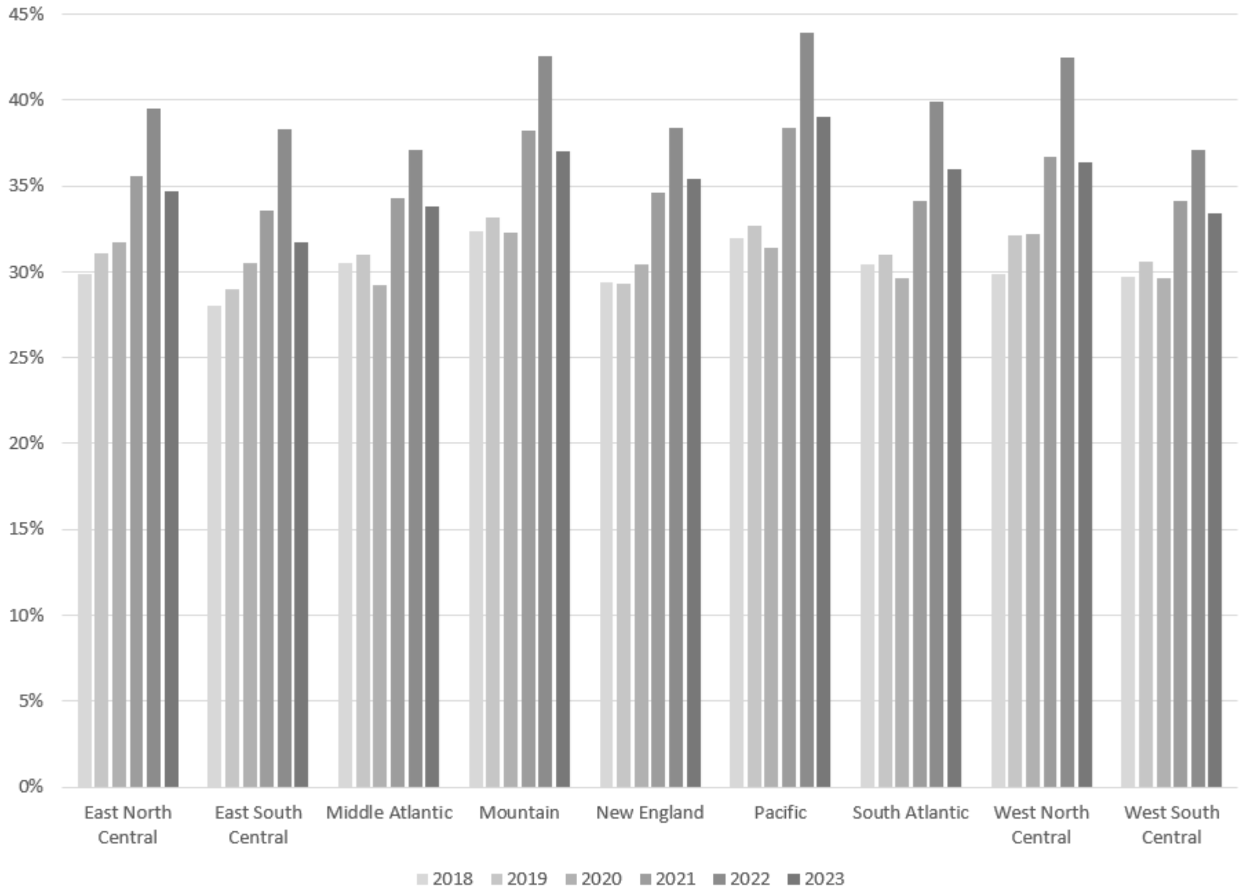

© 2025 Mohr DC et al. *JAMA Network Open*.

eFigure 4. Professional Stress Trends by Geography in the VHA

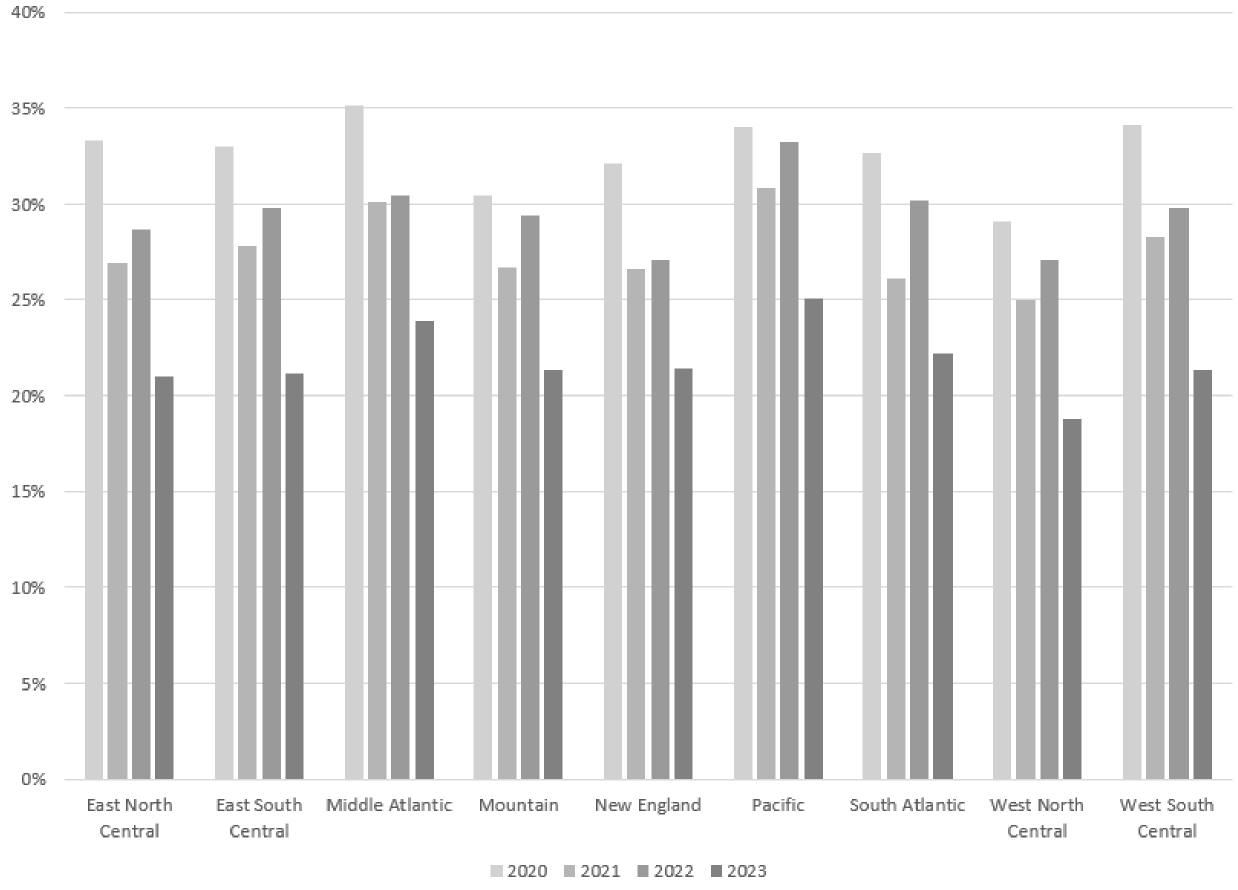

© 2025 Mohr DC et al. *JAMA Network Open*.
