# Supplementary material for: Burnout Trends Among US Health Care Workers
Source: JAMA Netw Open. 2025 Apr 21;8(4):e255954. doi: 10.1001/jamanetworkopen.2025.5954 (PMC12013355; doi:10.1001/jamanetworkopen.2025.5954)
Supplement: Supplement 2. — Data Sharing Statement [file jamanetwopen-e255954-s002.pdf]

Data Sharing Statement

Mohr. Burnout Trends Among US Health Care Workers. *JAMA Netw Open*. Published April 21, 2025. doi:10.1001/jamanetworkopen.2025.5954

Data
Data available: No

Additional Information
Explanation for why data not available: data available on Federal websites, contact authors for details
